# Comment on “Glycated hemoglobin and body mass index as mediators of GLP‐1RAs and Alzheimer's disease and related dementias in patients with type 2 diabetes”

**DOI:** 10.1002/alz.70295

**Published:** 2025-05-19

**Authors:** Xiangyu Zhu, Yongzhi Xie

**Affiliations:** ^1^ Department of Radiology The Third Xiangya Hospital Central South University Changsha China

**Keywords:** Alzheimer's disease, cognitive assessment, dynamic risk prediction

1

Dear Editor,

We read with great interest the article titled “Glycated hemoglobin and body mass index as mediators of GLP‐1RAs and Alzheimer's disease and related dementias in patients with type 2 diabetes” published in *Alzheimer's & Dementia* ^1^ and commend the authors for their rigorous methodological approach, including an active comparator new‐user design, to investigate the relationship between glucagon‐like peptide‐1 receptor agonists (GLP‐1RAs) and Alzheimer's disease and related dementias (ADRD). This work provides valuable insights into whether the reductions in glycated hemoglobin (HbA1c) and body mass index (BMI) partly explain the association between GLP‐1RAs and lower ADRD risk in individuals with type 2 diabetes (T2D). From a clinical perspective, however, we would like to share several concerns and considerations that may merit further exploration.

First, although the study design is commendable, the mean follow‐up for GLP‐1RA users (3.69 years) and other glucose‐lowering drug (GLD) users (4.86 years) may still be relatively short when evaluating a slowly progressive disease such as Alzheimer's. Longer‐term follow‐up could reveal more definitive trends in ADRD incidence and better characterize whether the observed protective effect persists or changes over time.

Second, the findings suggest that GLP‐1RAs have a substantial direct effect on lowering ADRD risk, with only a small proportion mediated through HbA1c or BMI reduction. Although this highlights an intriguing potential for GLP‐1RAs to act through alternative neuroprotective mechanisms (e.g., anti‐inflammatory or anti‐amyloid pathways), it also raises questions about the modest magnitude of mediation. Future research that integrates additional biomarker data (e.g., lipid levels, inflammatory markers, or neuroimaging) could help clarify these pathways and strengthen mechanistic inferences.

Third, the authors calculated E‐values for HbA1c (1.60) and BMI (1.56) to address possible unmeasured confounding. Despite these E‐values suggesting that a moderately strong confounder would be required to eliminate the observed association, certain clinical nuances—such as severity of comorbidities, medication adherence, or socio‐behavioral factors—might remain unaccounted for. Incorporating additional covariates or complementary analytical methods (e.g., propensity score calibration) could further reduce residual confounding.

Fourth, the study population derived from a single clinical research network may not fully mirror the heterogeneity of T2D populations in diverse healthcare settings. Variations in prescribing patterns, access to GLP‐1RAs, and clinical management of comorbidities can differ substantially. Wider adoption of multi‐center or international datasets could bolster external validity and ascertain whether these findings extend to other clinical contexts.

Lastly, older adults with long‐standing T2D frequently present with multiple comorbidities, and cognitive impairment can be subtle yet progressive. Stratifying analyses by age group and duration of diabetes, as well as evaluating more specific stages of cognitive decline (e.g., mild cognitive impairment), may help clarify which subsets of patients derive the most pronounced benefit from GLP‐1RA therapy.^2^

## AUTHOR CONTRIBUTIONS

Xiangyu Zhu, Yongzhi Xie: Conceived the ideas and framework for the letter, reviewed the article thoroughly, and drafted the initial manuscript. Yongzhi Xie: Contributed to the critical analysis of the article, provided insights into clinical applicability, and assisted in revising the draft for clarity and impact. All authors read and approved the final manuscript for submission.

## CONFLICT OF INTEREST STATEMENT

The authors declare no conflicts of interest. Author disclosures are available in the Supporting Information.

## Supporting information



Supporting Information

## Data Availability

Not applicable.
